# Gamma knife surgery for brain metastases from ovarian cancer

**DOI:** 10.1007/s00701-012-1376-3

**Published:** 2012-05-16

**Authors:** Akiyoshi Ogino, Tatsuo Hirai, Takao Fukushima, Toru Serizawa, Takao Watanabe, Atsuo Yoshino, Yoichi Katayama

**Affiliations:** 1 Department of Neurological Surgery, Nihon University School of Medicine, 30-1 Oyaguchi-Kamimachi, Itabashi-ku, Tokyo, 173-8610 Japan; 2 Tokyo Gamma Unit Center, Tsukiji Neurological Clinic, Tokyo, Japan

**Keywords:** Ovarian cancer, Brain metastases, Gamma knife surgery, Tumor control rate

## Abstract

**Background:**

Brain metastases from ovarian cancer are rare, but their incidence is increasing. The purpose of this study was to investigate the characteristics of brain metastases from ovarian cancer, and to assess the efficacy of treatment with gamma knife surgery (GKS).

**Methods:**

A retrospective review was performed of patients with brain metastases from ovarian cancer who were treated at the Tokyo Gamma Unit Center from 2006 to 2010.

**Results:**

Sixteen patients were identified. Their median age at diagnosis of brain metastases was 56.5 years, the median interval from diagnosis of ovarian cancer to brain metastases was 27.5 months, and the median number of brain metastases was 2. The median Karnofsky Performance Score (KPS) at the first GKS was 80. The median survival following diagnosis of brain metastases was 12.5 months, and 6-month and 1-year survival rates were 75 % and 50 %, respectively. The tumor control rate was 86.4 %. The KPS (<80 vs ≥80) and total volume of brain metastases (<10 cm^3^ vs ≥10 cm^3^) were significantly associated with survival according to a univariate analysis (*p* = 0.004 and *p* = 0.02, respectively).

**Conclusions:**

The results of this study suggest that GKS is an effective remedy and acceptable choice for the control of brain metastases from ovarian cancer.

## Introduction

Ovarian cancer is one of the most frequently diagnosed gynecological malignancies [17]. Because symptoms are usually not noticed in the early stages, ovarian cancer tends to be diagnosed in the advanced stage and the prognosis, therefore, is generally poor. Brain metastases from ovarian cancer are rare, with their incidence ranging from 0.49 % to 6.1 % [3, 6, 7, 9, 14, 15, 20–22, 24, 25, 34, 37]. However, recent studies have reported an increased incidence of brain metastases from ovarian cancer [6, 15, 21]. The primary reason for this increase is considered to be improvements in chemotherapy, which have led to better control of the primary malignancy and thus prolonged the life of the patients. In addition, early detection has been enabled by developments in imaging technologies [4, 12, 26]. Because gamma knife surgery (GKS) for brain metastases has a high local control rate and is minimally invasive [1, 2], it is generally an effective remedy. Nevertheless, to our knowledge, there have only been a few case reports and small series studies of GKS for brain metastases from ovarian cancer, and its efficacy remains unknown. In this study, we investigated the characteristics of patients with brain metastases from ovarian cancer, assessed the efficacy of GKS for local tumor control, and analyzed prognostic factors affecting patient survival.

## Materials and methods

### Patients

From March 2006 to January 2010, a total of 2603 patients with brain metastases were treated with GKS at the Tokyo Gamma Unit Center. The hospital’s database was searched to identify those with brain metastases from ovarian cancer, and the 16 identified patients were found to have been treated with GKS a total of 31 times. One hundred and nineteen tumors were treated in 31 GKS operations. Diagnoses in all patients were made with gadolinium-enhanced magnetic resonance imaging (MRI) with or without contrast-enhanced computed tomography.

Stereotactic radiosurgery was performed with a Leksell Gamma Knife model C (Elekta Instruments, Stockholm). All patients underwent thin-slice gadolinium-enhanced MRI after placement of the Leksell Model G stereotactic frame (Elekta Instruments) and the treatment plans were created using GammaPlan (Elekta Instruments). The dose delivered to the margin of the tumor ranged from 10.1 to 22.32 Gy (median 20.0 Gy). The maximum tumor dose ranged from 20.2 to 43.0 Gy (median 39.2 Gy).

After GKS, follow-up MRI was performed every 1–3 months. We assessed changes in tumor size with follow up MRI, and evaluated response to treatment with the Response Evaluation Criteria in Solid Tumors guideline [41]. At the 3 months following GKS, 66 of 119 tumors could be evaluated for tumor size, enabling an analysis of the relationship between tumor size and tumor control.

To assess the predictive factors for survival following diagnosis of brain metastases, the following characteristics were reviewed: age at diagnosis of brain metastases (<60 years vs ≥60 years), interval from diagnosis of ovarian cancer to brain metastases (<2 years vs ≥2 years), number of brain metastases at first GKS (single vs multiple), total volume of brain metastases at first GKS (<10 cm^3^ vs ≥10 cm^3^), Karnofsky Performance Score (KPS) at first GKS (<80 vs ≥80), and treatment for brain metastases (GKS alone vs combination therapy).

### Statistical analysis

Statistical analysis was performed with a personal computer running Stat View J-5.0 software (Abacus Concepts, Berkeley, CA, USA). Survival was calculated from the date of initial diagnosis of brain metastases until the date of death or last contact. The Kaplan–Meier method was used to calculate survival distributions. Differences in survival were analyzed using a log-rank test; *p* < 0.05 was considered significant.

## Results

From March 2006 to January 2010, a total of 2,603 patients with brain metastases were treated with GKS at the Tokyo Gamma Unit Center; 16 (0.6 %) of these patients had brain metastases from ovarian cancer. Two patients were treated with whole-brain radiotherapy (WBRT) before GKS (cases 4 and 9), two underwent surgical resection before GKS (cases 6 and 10), one was treated with WBRT and surgical resection before GKS (case 12), and one was treated with stereotactic radiotherapy before GKS (case 5). Patient characteristics of 16 patients are shown in Table 1.

**Table 1:** Patient characteristics of 16 patients with 119 brain metastases

Case no.	Age (years)	KPS at first GKS	Number of brain metastases at first GKS	Total volume of brain metastases at first GKS (cm^3^)	Symptoms	Pathologic diagnosis	Interval to brain metastases (months)	Other metastases	Survival following diagnosis of brain metastases (months)
1	51	80	2	8.5	None^a^	CCT	43	Liver, bone, AG	17
2	58	70	18	19.5	EW	NA	16	Lung	6
3	50	50	5	14.2	Seizure	ST	27	Lung, liver, bone	8
4	47	70	1	7.5	None^a^	ST	28	NA	11
5	38	100	2	2.3	EW^b^	NA	84	Colon, SL	13
6	58	50	4	22.5	Seizure	NA	34	NA	14
7	48	60	8	25.2	Aphasia	CCT	9	Lung	5
8	54	100	1	1.4	None^a^	NA	15	Lung, liver	16
9	73	90	2	1.6	EW	NA	15	Colon, spleen	31
10	41	100	1	9.9	None^a^	NA	42	None	34
11	71	80	13	13.6	Headache	CS	40	NA	6
12	61	100	1	9.5	EW	CS	25	NA	24
13	63	60	1	8.5	Dysarthria	NA	23	Colon, spleen, pancreas	12
14	52	60	3	5.8	EW	NA	72	Lung	4
15	67	80	4	42.6	Dizziness	ST	36	NA	11
16	46	100	1	0.6	None^a^	ST	0	SL	17

*EW* extremity weakness, *AG* adrenal gland, *SL* supraclavicular lymph node, *NA* not acquired, *CCT* clear cell tumor, *ST* serous tumor, *CS* carcinosarcoma

^a^Brain metastasis detected after MRI and/or CT

^b^Transient EW

The median age of the patients diagnosed with ovarian cancer and brain metastases was 53 years (range, 38–73 years) and 56.5 years (range, 44–75 years), respectively. The median interval from diagnosis of ovarian cancer to brain metastases was 27.5 months (range, 0–84 months), and the median number of brain metastases was 2 (range, 1–18). At the time of first GKS, the median KPS was 80 (range, 50–100).

At last follow-up, six patients were alive and ten patients had died. The overall median survival from the time of first GKS was 9.5 months (range, 3–29 months), from diagnosis of brain metastases was 12.5 months (range, 4–34 months), and from diagnosis of ovarian cancer was 46 months (range, 14–97 months). The 6-month and 1-year survival rates from the time of diagnosis of brain metastases were 75 % and 50 %, respectively. The 6-month and 1-year survival rates from the time of first GKS were 69 % and 31 %, respectively.

At 3 months following GKS, 66 tumors could be evaluated for tumor size; 17 tumors were in complete remission (CR), 40 showed partial response (PR), 8 stable disease, and 1 progressive disease (PD). The tumor of PD became a PR by performing additional GKS. The tumor control rate ([CR + PR]/total number of tumors × 100) was 86.4 %. When tumors were classified by size, namely <10 mm, 10–20 mm, 20–30 mm, and >30 mm, the tumor control rates were 89.7 %, 86.7 %, 80.0 %, and 50.0 %, respectively. No tumor hemorrhaged after GKS.

A total of nine patients (56.3 %) were found to have new brain metastases during the follow-up period. The interval between the date receiving initial GKS and the date of appearance of a new distant lesion was calculated. The median new distant-lesion-free survival period was 4.5 months (range, 1–15 months).

Age at diagnosis of brain metastases, interval to brain metastases, number of brain metastases at first GKS, and treatment for brain metastases were not associated with survival following brain metastases. The KPS at first GKS and total tumor volume at first GKS were associated with survival and were important predictors of survival according to a univariate analysis (*p* = 0.004 and *p* = 0.02, respectively). The median overall survival for the nine patients with KPS ≥80 was significantly longer than that of the seven patients with KPS <80 (17.0 vs 8.0 months, *p* = 0.004). The median overall survival for the ten patients with total volume of brain metastases <10 cm^3^ was significantly longer than that of the six patients with total volume of brain metastases ≥10 cm^3^ (16.5 vs 7.0 months, *p* = 0.02). The number of brain metastases was not significantly associated with survival, but the median overall survival for the six patients with a single metastases tended to be longer than that of the ten patients with multiple metastases (16.5 vs 9.5 months, *p* = 0.05) (Table 2).

**Table 2:** Univariate analysis of survival following diagnosis of brain metastases

Prognostic factor	Number of patients	Median survival (months)	Log-rank *p* value
Age at diagnosis of brain metastases			
<60 years	10	12.0	0.1841
≥60 years	6	13.0	
Interval to brain metastases			
<2 years	6	14.0	0.5592
≥2 years	10	12.0	
Number of brain metastases at first GKS			
Single	6	16.5	0.0507
Multiple	10	9.5	
Total volume of brain metastases at first GKS			
<10 cm^3^	10	16.5	0.0203
≥10 cm^3^	6	7.0	
KPS at first GKS			
<80	7	8.0	0.0042
≥80	9	17.0	
Treatment for brain metastases			
GKS alone	10	9.5	0.2020
Combination therapy	6	19.0	

## Discussion

Ovarian cancer is one of the most frequent causes of malignancy in women [17]. The majority of patients are discovered with advanced disease because very few symptoms are observed in the early stages of ovarian cancer. Brain metastases from ovarian cancer is uncommon—its incidence ranges from 0.49 % to 6.1 % [3, 6, 7, 9, 14, 15, 20–22, 24, 25, 34, 37]. Pectasides et al. [35] reported an estimated incidence of 1.01 % in a review of 22,240 patients with ovarian cancer. However, recent studies have suggested that the incidence of brain metastases from ovarian cancer is increasing [6, 15, 21]. Platinum-based chemotherapy has led to improved survival rates among ovarian cancer patients, but these agents have poor blood–brain barrier permeability and brain metastases have thus become a late manifestation of ovarian cancer. The development of better imaging techniques has also enabled diagnosis on the basis of smaller brain metastatic lesions [4, 12, 26].

In the review of brain metastases from ovarian cancer by Pectasides et al. [35], single brain metastases occurred in 43.8 % of patients, multiple metastases in 51.6 %, and leptomeningeal metastases alone in 4.6 %. In this study, 37.5 % of patients had single metastases, 62.5 % had multiple metastases, and no patient had leptomeningeal metastases.

Data from the literature indicate that the median age at diagnosis of ovarian cancer ranges from 51 to 59.5 years, and the median age at diagnosis of brain metastases ranges from 52.5 to 58 years. The median interval to brain metastases ranges from 14.5 to 46 months [3, 5–7, 9, 14, 15, 20–22, 24, 25, 34, 37] (Tables 3, 4). The results from our patients support these data.

**Table 3:** Literature review of brain metastases from ovarian cancer

Authors	Study period	Number of patients	Percentage incidence	Median age at the diagnosis of ovarian cancer	Median age at the diagnosis of brain metastases	Median interval to brain metastases	Median survival following brain metastases
Anupol et al. [3]	1986–2000	15	1.4^a^	58.0^c^	-	22.0	6.0
Chen et al. [5]	1985–2002	19	1.0^b^	51.0	54.0	25.2	16.3
Chen et al. [6]	2000–2007	10	1.9^a^	56.6^c^	58.7^c^	21.0	3.0
Cohen et al. [7]	1975–2001	72	0.9^a^	50.4^c^	53.7^c^	22.08	6.27
Corn et al. [9]	1965–1994	32	0.8^a^	-	56.0	24.0	4.0
Gadducci et al. [14]	1999–2005	12	6.1^a^	59.5	-	33.5	8.3
Geisler and Geisler [15]	1979–1992	16	3.3^a^	56.6^c^	-	19.0	3.0
Kim et al. [20]	1995–2005	13	2.7^a^	52.0	55.0	28.0	7.0
Kolomainen et al. [21]	1980–2000	18	0.49^a^	52.0	57.0	46.0	7.0
Kumar et al. [22]	1991–2001	18	2.7^a^	-	54.0	29.0	4.0
Lee et al. [24]	1983–2005	18	1.3^a^	55.0	56.0	28.0	14.0
LeRoux et al. [25]	1980–1989	14	1.1^a^	-	52.5	14.5	3.0
Pectasides et al. [34]	1983–2004	17	1.17^a^	-	58.0	15.9	5.7
Rodriguez et al. [37]	1977–1990	15	1.9	-	57.0	18.5	9.0
This study	2006–2010	16	0.6^b^	53.0	56.5	27.5	12.5

*Surg* surgical resection, *C/T* chemotherapy, *SRS* stereotactic radiosurgery

^a^Percentage incidence of ovarian cancer

^c^Mean age

^b^Percentage incidence of brain metastases

^c^Mean age

**Table 4:** Literature review of brain metastases from ovarian cancer: treatments

Authors	Treatment for brain metastases, *n* (%)									
	WBRT	Surg + WBRT	Surg alone	Surg + C/T + WBRT	C/T + WBRT	WBRT + SRS	SRS alone	Surg + SRS	Surg + WBRT + SRS	Others
Anupol et al. [3]	3 (20)	1 (7)	0 (0)	5 (33)	4 (27)	0 (0)	0 (0)	0 (0)	0 (0)	2 (13)
Chen et al. [5]	9 (47)	6 (32)	1 (5)	0 (0)	0 (0)	1 (5)	0 (0)	2 (11)	0 (0)	0 (0)
Chen et al. [6]	3 (30)	0 (0)	0 (0)	1 (10)	4 (40)	0 (0)	0 (0)	0 (0)	0 (0)	2 (20)
Cohen et al. [7	35 (51)	12 (17)	8 (12)	0 (0)	0 (0)	1 (1)	0 (0)	1 (1)	0 (0)	12 (17)
Corn et al. [9]	24 (75)	0 (0)	0 (0)	0 (0)	5 (16)	3 (9)	0 (0)	0 (0)	0 (0)	0 (0)
Gadducci et al. [14]	3 (25)	1 (8)	1 (8)	0 (0)	1 (8)	0 (0)	2 (17)	0 (0)	0 (0)	4 (33)
Geisler and Geisler [15]	8 (50)	5 (31)	0 (0)	0 (0)	2 (13)	0 (0)	0 (0)	0 (0)	0 (0)	1 (6)
Kim et al. [20]	2 (15)	0 (0)	0 (0)	1 (8)	3 (23)	4 (31)	1 (8)	0 (0)	1 (8)	1 (8)
Kolomainen et al. [21]	6 (33)	1 (6)	0 (0)	0 (0)	0 (0)	0 (0)	0 (0)	0 (0)	0 (0)	11 (61)
Kumar et al. [22]	8 (44)	0 (0)	0 (0)	4 (22)	5 (28)	0 (0)	0 (0)	0 (0)	0 (0)	1 (6)
Lee et al. [24]	8 (50)	0 (0)	0 (0)	0 (0)	0 (0)	0 (0)	7 (44)	0 (0)	0 (0)	1 (6)
LeRoux et al. [25]	8 (57)	5 (35)	0 (0)	0 (0)	0 (0)	0 (0)	0 (0)	0 (0)	0 (0)	1 (7)
Pectasides et al. [34]	4 (24)	0 (0)	0 (0)	2 (12)	6 (35)	0 (0)	0 (0)	0 (0)	0 (0)	5 (29)
Rodriguez et al. [37]	4 (27)	1 (7)	0 (0)	3 (20)	6 (40)	0 (0)	0 (0)	0 (0)	0 (0)	1 (7)
This study	0 (0)	0 (0)	0 (0)	0 (0)	0 (0)	2 (13)	10 (63)	2 (13)	1 (6)	1 (6)

*Surg* surgical resection, *C/T* chemotherapy, *SRS* stereotactic radiosurgery

^a^Percentage incidence of ovarian cancer

^c^Mean age

^b^Percentage incidence of brain metastases

^c^Mean age

Brain metastases from ovarian cancer represent a late manifestation; once brain metastases occur, survival is generally poor, regardless of treatment [14, 21]. Therapies for brain metastases include surgical resection, radiotherapy, chemotherapy, or their combination. Cohen et al. [7] reported that the combination of surgical resection and WBRT resulted in longer survival than WBRT alone or surgical resection alone (median survival of 23 months, 5 months, and 7 months, respectively). Anupol et al. [3] reported that combined treatment with radiation and surgical resection with or without chemotherapy led to a good prognosis in patients with brain metastases. Other authors have also supported an aggressive combination therapy approach [11, 15, 18, 22, 25, 34, 36, 37]. In our study, treatment for brain metastases was not significantly associated with survival, but the median overall survival for the six patients treated with combination therapy tended to be longer than that of the ten patients treated GKS alone (19.0 vs 9.5 months, *p* = 0.2). Our result also supports combination therapy.

The efficacy of systemic chemotherapy for brain metastases from ovarian cancer remains controversial. Cooper et al. [8] reported a response in three patients with brain metastases treated with carboplatin, Melichar et al. [27] reported a response in a patient treated with cisplatin and gemcitabine, and Watanabe et al. [42] reported a response in a patient treated with carboplatin and docetaxel. To our knowledge, the only literature providing support for the efficacy of chemotherapy for brain metastases from ovarian cancer are case reports and small case series.

GKS for brain metastases has become increasingly common because GKS is a noninvasive modality that provides good local control [1, 2]. Muacevic et al. [30] reported that there were no significant difference in a 1-year local tumor control rate and 1-year survival rate between patients treated with GKS alone for solitary brain metastases and those who were treated with surgical resection plus WBRT. O’Neil et al. [32] reported that there was no significant difference in the 1-year survival rate between patients treated with GKS and those who underwent surgical resection (62 % vs 56 %), and GKS resulted in significantly better local control compared with surgical resection (recurrence rate of 0 % vs 58 %).

GKS for brain metastases from ovarian cancer was first reported by Kawana et al. [19] in 1997. They described a case of multiple brain metastases successfully treated by a multimodality treatment including GKS. Lee et al. [24] reported that GKS for brain metastases from ovarian cancer resulted in longer survival compared with WBRT (median survival of 29 vs 6 months), and Corn et al. [10] reported that radiosurgery led to a more frequent complete remission (40 % vs 29 %), and higher 2-year survival rate (60 % vs 15 %) than WBRT alone. Kim et al. [20] reported that treatment modalities including GKS were associated with survival and that patients with brain metastases had better outcomes with GKS. However, these reports are all from small series of patients and no prospective studies of GKS for brain metastases from ovarian cancer have been conducted. Our study is a retrospective study, but it is the second largest study of GKS for brain metastases from ovarian cancer to be performed. Monaco et al. [28] reported on 27 patients with brain metastases from ovarian cancer and endometrial carcinoma treated with GKS. The median survival after brain metastases was 7 months and the 1-year survival rate was 22 %. All tumors were controlled. They suggested that GKS was an acceptable choice for brain metastases, but their report included the results of the six patients with brain metastases from endometrial carcinoma.

In our study, the tumor control rate was 86.4 % and no tumors hemorrhaged after GKS. Our results show tumor control rates comparable to those of lung cancers (72.7–94 %) [16, 33, 38], breast cancers (90–94 %) [13, 23, 31], and renal cell cancer (82.6–96 %) [29, 39, 40]. GKS for brain metastases from ovarian cancer provides good local control with few side effects.

Several reports have identified predictive factors for brain metastases from ovarian cancer. KPS, number of brain metastases, recursive partitioning analysis, and extracranial metastases have all been associated with survival [3, 5, 9, 18, 20, 25]. In our study, the KPS ≥80 % and total volume of brain metastases <10 cm^3^ were factors to be significantly associated with longer survival (*p* = 0.004 and *p* = 0.02, respectively). Age at diagnosis of brain metastases, interval to brain metastases, number of brain metastases, and treatment for brain metastases were not associated with survival. However, patients with a single metastases tended to survive longer than patients with multiple metastases (*p* = 0.05).

Only a limited number of patients with brain metastases from ovarian cancer have been treated with GKS to date, and prospective data are lacking. However, the results of this study indicate that GKS for brain metastases from ovarian cancer provides good local control with few side effects. GKS thus appears to be an acceptable choice for the control of brain lesions in ovarian cancer patients. Combination therapy improves prognosis for patient with brain metastases from ovarian cancer. GKS can become one modality of combination therapy.

## Conclusions

Brain metastases from ovarian cancer are rare, but their incidence is increasing as patient survival has been extended by successful platinum-based chemotherapy and improved imaging techniques have enabled the identification of smaller lesions. In our study, the median survival from brain metastases was 12.5 months, and the local control rate was 86.4 %. The KPS and total volume of brain metastases were important factors predictive of survival. Our results suggest that GKS is an acceptable therapy for brain metastases from ovarian cancer.

## References

[1] Adler JR, Cox RS, Kaplan I, Martin DP (1992). Stereotactic radiosurgical treatment of brain metastases. J Neurosurg.

[2] Alexander E, Moriarty TM, Davis RB, Wen PY, Fine HA, Black PM, Kooy HM, Loeffler JS (1995). Stereotactic radiosurgery for the definitive, noninvasive treatment of brain metastases. J Natl Canc Inst.

[3] Anupol N, Ghamande S, Odunsi K, Driscoll D, Lele S (2002). Evaluation of prognostic factors and treatment modalities in ovarian cancer patients with brain metastases. Gynecol Oncol.

[4] Bruzzone M, Campora E, Chiara S, Giudici S, Merlini L, Simoni C, Mammoliti S, Rubagotti A, Rosso R (1993). Cerebral metastases secondary to ovarian cancer: still an unusual event. Gynecol Oncol.

[5] Chen PG, Lee SY, Barnett GH, Vogelbaum MA, Saxton JP, Fleming PA, Suh JH (2005). Use of the Radiation Therapy Oncology Group recursive partitioning analysis classification system and predictors of survival in 19 women with brain metastases from ovarian carcinoma. Cancer.

[6] Chen YL, Cheng WF, Hsieh CY, Chen CA (2011). Brain metastasis as a late manifestation of ovarian carcinoma. Eur J Canc Care (Engl).

[7] Cohen ZR, Suki D, Weinberg JS, Marmor E, Lang FF, Gershenson DM, Sawaya R (2004). Brain metastases in patients with ovarian carcinoma: prognostic factors and outcome. J Neurooncol.

[8] Cooper KG, Kitchener HC, Parkin DE (1994). Cerebral metastases from epithelial ovarian carcinoma treated with carboplatin. Gynecol Oncol.

[9] Corn BW, Greven KM, Randall ME, Wolfson AH, Kim RY, Lanciano RM (1995). The efficacy of cranial irradiation in ovarian cancer metastatic to the brain: analysis of 32 cases. Obstet Gynecol.

[10] Corn BW, Mehta MP, Buatti JM, Wolfson AH, Greven KM, Kim RY, Dunton CJ, Loeffler JS (1999). Stereotactic Irradiation: potential new treatment method for brain metastases resulting from ovarian cancer. Am J Clin Oncol.

[11] D’Andrea G, Roperto R, Dinia L, Caroli E, Salvati M, Ferrante L (2005). Solitary cerebral metastases from ovarian epithelial carcinoma: 11 cases. Neurosurg Rev.

[12] Dauplat J, Nieberg RK, Hacker NF (1987). Central nervous system metastases in epithelial ovarian carcinoma. Cancer.

[13] Firlik KS, Kondziolka D, Flickinger JC, Lunsford LD (2000). Stereotactic radiosurgery for brain metastases from breast cancer. Ann Surg Oncol.

[14] Gadducci A, Tana R, Teti G, Fanucchi A, Pasqualetti F, Cionini L, Genazzani AR (2007). Brain recurrences in patients with ovarian cancer: report of 12 cases and review of the literature. Anticancer Res.

[15] Geisler JP, Geisler HE (1995). Brain metastases in epithelial ovarian carcinoma. Gynecol Oncol.

[16] Jawahar A, Matthew RE, Minagar A, Shukla D, Zhang JH, Willis BK, Ampil F, Nanda A (2004). Gamma knife surgery in the management of brain metastases from lung carcinoma: a retrospective analysis of survival, local tumor control, and freedom from new brain metastasis. J Neurosurg.

[17] Jemal A, Siegel R, Xu J, Ward E (2010). Cancer statistics, 2010. CA Cancer J Clin.

[18] Kaminsky-Forrett MC, Weber B, Conroy T, Spaeth D (2000). Brain metastases from epithelial ovarian carcinoma. Int J Gynecol Cancer.

[19] Kawana K, Yoshikawa H, Yokota H, Onda T, Nakagawa K, Tsutsumi O, Taketani Y (1997). Successful treatment of brain metastases from ovarian cancer using gamma-knife radiosurgery. Gynecol Oncol.

[20] Kim TJ, Song S, Kim CK, Kim WY, Choi CH, Lee JH, Lee JW, Bae DS, Kim BG (2007). Prognostic factors associated with brain metastases from epithelial ovarian carcinoma. Int J Gynecol Cancer.

[21] Kolomainen DF, Larkin JM, Badran M, A'Hern RP, King DM, Fisher C, Bridges JE, Blake PR, Barton DP, Shepherd JH, Kaye SB, Gore ME (2002). Epithelial ovarian cancer metastasizing to the brain: a late manifestation of the disease with an increasing incidence. J Clin Oncol.

[22] Kumar L, Barge S, Mahapatra AK, Thulkar S, Rath GK, Kumar S, Mishra R, Dawar R, Singh R (2003). Central nervous system metastases from primary epithelial ovarian cancer. Cancer Control.

[23] Lederman G, Wronski M, Fine M (2001). Fractionated radiosurgery for brain metastases in 43 patients with breast carcinoma. Breast Cancer Res Treat.

[24] Lee YK, Park NH, Kim JW, Song YS, Kang SB, Lee HP (2008). Gamma-knife radiosurgery as an optimal treatment modality for brain metastases from epithelial ovarian cancer. Gynecol Oncol.

[25] LeRoux PD, Berger MS, Elliott JP, Tamimi HK (1991). Cerebral metastases from ovarian carcinoma. Cancer.

[26] McMeekin DS, Kamelle SA, Vasilev SA, Tillmanns TD, Gould NS, Scribner DR, Gold MA, Guruswamy S, Mannel RS (2001). Ovarian cancer metastatic to the brain: what is the optimal management?. J Surg Oncol.

[27] Melichar B, Urminska H, Kohlova T, Nova M, Cesak T (2004). Brain metastases of epithelial ovarian carcinoma responding to cisplatin and gemcitabine combination chemotherapy: a case report and review of the literature. Gynecol Oncol.

[28] Monaco E, Kondziolka D, Mongia S, Niranjan A, Flickinger JC, Lunsford LD (2008). Management of brain metastases from ovarian and endometrial carcinoma with stereotactic radiosurgery. Cancer.

[29] Mori Y, Kondziolka D, Flickinger JC, Logan T, Lunsford LD (1998). Stereotactic radiosurgery for brain metastasis from renal cell carcinoma. Cancer.

[30] Muacevic A, Kreth FW, Horstmann GA, Schmid-Elsaesser R, Wowra B, Steiger HJ, Reulen HJ (1999). Surgery and radiotherapy compared with gamma knife radiosurgery in the treatment of solitary cerebral metastases of small diameter. J Neurosurg.

[31] Muacevic A, Kreth FW, Tonn JC, Wowra B (2004). Stereotactic radiosurgery for multiple brain metastases from breast carcinoma. Cancer.

[32] O’Neill BP, Iturria NJ, Link MJ, Pollock BE, Ballman KV, O'Fallon JR (2003). A comparison of surgical resection and stereotactic radiosurgery in the treatment of solitary brain metastases. Int J Radiat Oncol Biol Phys.

[33] Pan HC, Sheehan J, Stroila M, Steiner M, Steiner L (2005). Gamma knife surgery for brain metastases from lung cancer. J Neurosurg.

[34] Pectasides D, Aravantinos G, Fountzilas G, Kalofonos C, Efstathiou E, Karina M, Pavlidis N, Farmakis D, Economopoulos T, Dimopoulos MA (2005). Brain metastases from epithelial ovarian cancer. The Hellenic Cooperative Oncology Group (HeCOG) experience and review of the literature. Anticancer Res.

[35] Pectasides D, Pectasides M, Economopoulos T (2006). Brain metastases from epithelial ovarian cancer: a review of the literature. Oncologist.

[36] Pothuri B, Chi DS, Reid T, Aghajanian C, Venkatraman E, Alektiar K, Bilsky M, Barakat RR (2002). Craniotomy for central nervous system metastases in epithelial ovarian carcinoma. Gynecol Oncol.

[37] Rodriguez GC, Soper JT, Berchuck A, Oleson J, Dodge R, Montana G, Clarke-Pearson DL (1992). Improved palliation of cerebral metastases in epithelial ovarian cancer using a combined modality approach including radiation therapy, chemotherapy, and surgery. J Clin Oncol.

[38] Sheehan JP, Sun MH, Kondziolka D, Flickinger J, Lunsford LD (2002). Radiosurgery for non-small cell lung carcinoma metastatic to the brain: long-term outcomes and prognostic factors influencing patient survival time and local tumor control. J Neurosurg.

[39] Sheehan JP, Sun MH, Kondziolka D, Flickinger J, Lunsford LD (2003). Radiosurgery in patients with renal cell carcinoma metastasis to the brain: long-term outcomes and prognostic factors influencing survival and local tumor control. J Neurosurg.

[40] Shuto T, Inomori S, Fujino H, Nagano H (2006). Gamma knife surgery for metastatic brain tumors from renal cell carcinoma. J Neurosurg.

[41] Therasse P, Arbuck SG, Eisenhauer EA, Wanders J, Kaplan RS, Rubinstein L, Verweij J, Van Glabbeke M, van Oosterom AT, Christian MC, Gwyther SG (2000). New guidelines to evaluate the response to treatment in solid tumors. European Organization for Research and Treatment of Cancer, National Cancer Institute of the United States, National Cancer Institute of Canada. J Natl Cancer Inst.

[42] Watanabe A, Shimada M, Kigawa J, Iba T, Oishi T, Kanamori Y, Terakawa N (2005). The benefit of chemotherapy in a patient with multiple brain metastases and meningitis carcinomatosa from ovarian cancer. Int J Clin Oncol.

